# Biophysical Characterisation of Calumenin as a Charged F508del-CFTR Folding Modulator

**DOI:** 10.1371/journal.pone.0104970

**Published:** 2014-08-13

**Authors:** Rashmi Tripathi, Nathalie Benz, Bridget Culleton, Pascal Trouvé, Claude Férec

**Affiliations:** 1 INSERM UMR1078, Brest, France; 2 Université de Bretagne Occidentale, Faculté de Medecine et des sciences de la santé, Brest, France; 3 Association Gaétan Saleün, Brest, France; 4 Department of Biology, National University of Ireland, Maynooth, Maynooth, Co Kildare, Ireland; 5 Hôpital Morvan, Laboratoire de Génétique Moléculaire et d‘Histocompatibilité, Brest, France; 6 Etablissement Français du Sang-Bretagne, Brest, France; University of South Florida College of Medicine, United States of America

## Abstract

The cystic fibrosis transmembrane regulator (CFTR) is a cyclic-AMP dependent chloride channel expressed at the apical surface of epithelial cells lining various organs such as the respiratory tract. Defective processing and functioning of this protein caused by mutations in the CFTR gene results in loss of ionic balance, defective mucus clearance, increased proliferation of biofilms and inflammation of human airways observed in cystic fibrosis (CF) patients. The process by which CFTR folds and matures under the influence of various chaperones in the secretory pathway remains incompletely understood. Recently, calumenin, a secretory protein, belonging to the CREC family of low affinity calcium binding proteins has been identified as a putative CFTR chaperone whose biophysical properties and functions remain uncharacterized. We compared hydropathy, instability, charge, unfoldability, disorder and aggregation propensity of calumenin and other CREC family members with CFTR associated chaperones and calcium binding proteins, wild-type and mutant CFTR proteins and intrinsically disordered proteins (IDPs). We observed that calumenin, along with other CREC proteins, was significantly more charged and less folded compared to CFTR associated chaperones. Moreover like IDPs, calumenin and other CREC proteins were found to be less hydrophobic and aggregation prone. Phylogenetic analysis revealed a close link between calumenin and other CREC proteins indicating how evolution might have shaped their similar biophysical properties. Experimentally, calumenin was observed to significantly reduce F508del-CFTR aggregation in a manner similar to AavLEA1, a well-characterized IDP. Fluorescence microscopy based imaging analysis also revealed altered trafficking of calumenin in bronchial cells expressing F508del-CFTR, indicating its direct role in the pathophysiology of CF. In conclusion, calumenin is characterized as a charged protein exhibiting close similarity with IDPs and is hypothesized to regulate F508del-CFTR folding by electrostatic effects. This work provides useful insights for designing optimized synthetic structural correctors of CFTR mutant proteins in the future.

## Introduction

Cystic fibrosis (CF) is the most common autosomal recessive genetic disorder affecting one in every 2000–3000 neonates in the Caucasian population, caused by loss of function mutations in the cystic fibrosis transmembrane regulator gene [1]. This gene encodes a chloride channel with two nucleotide binding domains (NBDs) 1 and 2, a regulatory domain (R) and the membrane spanning domains (MSDs) 1 and 2. The interfaces between NBDs and MSDs are formed by the cytoplasmic loops (CLs) 1–4. Individual domains form loosely folded conformations co-translationally, while post-translational processing results in the formation of higher ordered tertiary structures (for review see [2]). Its function as an ATP-gated ion channel following R-domain phosphorylation by cAMP-dependent protein kinase (PKA) is dependent on ATP binding at two composite sites (site 1 consisting of NBD1 Walker motifs and signature motif of NBD2 and site 2 comprising the NBD2 Walker motifs and the NBD1 signature motif [3]).

Nearly 2000 sequence variants of this gene have been identified so far in CF patients (Cystic Fibrosis Mutation Database:). These mutations have been grouped into six different classes and affect protein synthesis, trafficking, regulation, conductance, splicing or transcription and protein stability of the CFTR chloride channel respectively [4]. In the most commonly occurring F508del mutation, which accounts for nearly two thirds of mutated alleles in CF patients, native NBD-CL4 and CL1 interaction is disrupted, compromising CFTR domain assembly and maturation [2], [5], [6]. As a result, ATP-dependent relay of conformational changes of NBDs and MSDs involved in chloride channel gating are affected along with CFTR biogenesis [7]. Besides regulating chloride transport, CFTR loss of function also hampers other physiological processes regulated by this channel, such as sodium transport, ATP transport, vesicular transport, acidification of intracellular organelles and bicarbonate-chloride exchange [8]–[10]. The most common clinical symptoms of CF include chronic lung infection, pancreatic insufficiency, male infertility and reduced life expectancy (39 years at an average), with lung disease being the most common cause of morbidity in CF patients [11].

Recently, calumenin, belonging to the CREC (abbreviation for Cab45, reticulocalbin, ERC-45 and calumenin) family of low affinity calcium binding proteins containing multiple EF-hands, has been identified as a putative G551D-CFTR (Gly to Asp mutation at position 551) chaperone [12]. This CFTR mutation, located in the NBD, is known to cause gating defects [13] and is observed in approximately 5% of CF cases, with a severe clinical phenotype [11], [12]. Besides associating specifically with G551D-CFTR, calumenin is also predicted to interact with wild-type CFTR [12]. Evidence for this interaction has been provided using co-immunoprecipitation and surface plasmon resonance, which we believe is insufficient to conclude that the interaction between calumenin and CFTR is ‘direct’ in the cellular environment. Nevertheless, we assume that calumenin lies in close proximity to CFTR, or at least exists in the same protein complex and hypothesize that it might have an effect on CFTR folding. Besides calumenin, RCN1 and RCN2 are the other two CREC proteins that have been identified as interacting partners of wild-type and F508del-CFTR, with RCN1 being enriched in the wild-type CFTR associated proteome and RCN2 being enriched in both the wild-type and F508del-CFTR associated proteomes [14].

Currently, the biophysical properties and functions of calumenin as a chaperone remain unknown. The aim of our study was to investigate the biophysical features of the calumenin amino acid sequence, test its effects on the folding dynamics of F508del-CFTR protein in the presence of calcium, MgATP and ethylenediaminetetraacetic acid (EDTA) and analyze its intra-cellular distribution in human CFBE41o- bronchial cells expressing wild-type or F508del-CFTR. We analyzed various parameters (such as hydropathy, instability, charge, unfoldability, state of disorder and aggregation propensity) of calumenin, along with other CREC family members, CFTR associated chaperones (such as HSC70, HSP40, AHA1 etc.), calcium binding proteins (such as calgranulin, PSOR1, PDCD6), wild-type and mutant CFTR sequences (F508del-CFTR, G551D-CFTR) and five previously described intrinsically disordered proteins (IDPs) [15]–[17]. IDPs were included as a control group in our analysis, since they are characterized by a high state of disorder in their polypeptide sequences [15], [18]. This property, along with protein flexibility, has also been observed in some chaperones such as HSP70 and is believed to aid in their molecular recognition properties [19], [20].

We report that calumenin and other CREC family members are significantly more charged and less folded compared to conventional CFTR associated chaperones. In contrast to wild-type and mutant CFTR proteins, calumenin along with other CREC proteins, showed significantly less hydropathy and aggregation propensity. Phylogenetic analysis revealed common ancestry of CREC proteins and similarity with both chaperones and calcium binding proteins. In order to investigate the underlying molecular mechanism of calumenin action, *in vitro* protein-folding experiments were performed which demonstrated a reduction in F508del-CFTR aggregation by calumenin in the presence of calcium, MgATP and EDTA. Similar anti-aggregation properties were observed for AavLEA1, a well-characterized IDP described previously in literature [17], [21]. Imaging analysis revealed that in CFBE41o- cells expressing wild-type CFTR, calumenin demonstrated extensive localization in the endoplasmic reticulum (ER) and Golgi compartments. These results are consistent with its role as a secretory protein [22]. Unlike the CFTR [23], it was largely excluded from EEA1 containing endocytic vesicles suggesting that its association with CFTR might be restricted to specific intra-cellular compartments and that the two proteins might undergo dissociation during its secretion. Moreover, in a large proportion of cells expressing F508del-CFTR, it was translocated to the cytoplasm and the nucleus and exhibited increased retention in the endocytic pathway, indicating altered physiology of mutant cells. Overall, our results draw a correlation between the computationally predicted physicochemical similarity of calumenin with disordered proteins and their experimentally determined *in vitro* anti-aggregation activity. We also discuss the implications of these findings in designing optimized synthetic correctors of mutant CFTR proteins in the future.

## Materials and Methods

### Bioinformatics

Calumenin, along with other CFTR interacting proteins with chaperone functions, calcium binding properties or those belonging to the CREC family were selected from the dataset published by Wang et al. in 2006 [14]. We decided to incorporate two other CREC proteins RCN3 and SDF4 in the dataset for comparison as well. Five proteins previously described as being intrinsically disordered [15]–[17] along with wild-type CFTR and its mutant variants F508del-CFTR, G551DCFTR were also included. Measurements for various biophysical parameters such as hydropathy, given by GRAVY scores (Grand average of hydropathy), instability, unfoldability, charge, disorder (IUPRED scores) and aggregation propensity were performed for all the proteins using the ProtParam tool (http://web.expasy.org/protparam/) [24], FoldIndex (http://bip.weizmann.ac.uk.il/fldbin/findex) [25], IUPRED server (http://iupred.enzim.hu) [26], [27] and Aggrescan server (http://bioinf.uab.es/aggrescan/) [28]. IUPRED and aggregation scores were normalized for protein length. Table S1 shows the raw scores. Phylogenetic tree construction was performed after multiple sequence alignment using CLUSTAL omega [29], version 1.2.0, with five iterations (for guide tree construction and HMM profiling). Aligned sequences were used for tree construction in the PHYLIP format using CLUSTALW2 program with UPGMA clustering method, distance correction and percent identity matrix parameter being true [30].

### Statistical analysis

All statistical analysis was performed using R 3.0.1 statistical package. Data normality was assessed using the Shapiro-Wilk test and depending upon whether data was observed to be normal or not, ANOVA or Kruskal-Wallis test was performed to estimate whether the mean difference observed between different protein classes was significant enough. Post-hoc analysis was carried out using either the Tukey's test or Mann-Whitney test with Bonferroni correction. Principal component analysis and hierarchial clustering were performed to observe the correlations between different biophysical parameters and the distribution of proteins across the first two components that describe the maximum variation in the data. Principal component analysis (PCA) applies orthogonal transformation to convert a set of observations of possibly correlated variables into a set of values of uncorrelated variables called principal components. The number of principal components is less than or equal to the number of original variables. This transformation is defined in such a way that the first principal component has the largest possible variance and each succeeding component in turn has the highest variance possible under the constraint that it is orthogonal to (i.e., uncorrelated with) the preceding components. Hierarchial clustering further highlights the variation observed between various proteins on the PCA map, where the sum of the within-cluster inertia are calculated for each partition. The suggested partition is the one with the higher relative loss of inertia (i(clustersn+1)/i(cluster n)). The absolute loss of inertia (defined as the squared distance between clusters) (i(cluster n)-i(cluster n+1)) is plotted with the tree.

### Cloning of AavLEA1

The *AavLEA1* gene was cloned as described in Goyal *et al.* [31] with minor modifications at the NUI Maynooth, Ireland. Briefly, the gene was amplified from cDNA (prepared using SuperScript III RT (Invitrogen)) according to Goyal *et al.* and cloned into the pET15b vector (Novagen) on the *NdeI/BamHI* sites coding for AavLEA1 protein and a N-terminal His6 tag. The vector was then transformed into BL21 (DE3) cells. The plasmid has been deposited in Addgene plasmid reference database (reference number: 53093).

### F508del-CFTR anti-aggregation assay

Pure recombinant human calumenin was obtained from Prospec (Israel) and purified full length F508del-CFTR [32] was a generous gift from Prof. Robert Ford (University of Manchester, UK). AavLEA1 was purified using Ni-NTA Fast Start kit (Qiagen) from BL21 (DE3) *E.coli* cells containing pET15b-AavLEA1 according to the manufacturer's instructions and dialyzed extensively into water. Proteins were concentrated using Vivaspin6 protein concentrators, 3 kDa (Sartorius Stedim Biotech). Protein concentrations were determined by plotting a standard curve using different concentrations of bovine serum albumin and Folin's reagent, by measuring absorbance at 660 nm of reaction mixtures after incubation at 37°C for 20 minutes in the dark. F508del-CFTR protein aggregation assay to monitor formation of off-pathway species were initiated by rapid dilution of F508del-CFTR in 6 M GuHCl to a final concentration of 1 µM into pre-warmed (37°C) folding mix (Buffer R: 100 mM Tris-HCl, pH 7.4, 0.385 M L-arginine-HCl, 2 mM EDTA, 10 mM dithiothreitol and 25 mM GuHCl). In experiments containing Calumenin or AavLEA1, a final concentration of 0.5 µM of protein in Buffer C (20 mM imidazole, pH 7.0, 2 mM magnesium acetate, 10 mM (NH_4_)_2_SO_4_, 25 mM KCl, 1 mM dithiothreitol) was added. BSA was used as a negative control at 0.5 µM concentration. MgATP was used at a final concentration of 50 µM. CaCl_2_ was used at 0.75 mM and 2 mM final concentration. EDTA was used at a final concentration of 5 mM. Total reaction volume used was 100 µl. Aggregation was followed over time at 37°C by turbidity measured at 405 nm in a Biophotometer (eppendorf). Multiple measurements were made to ensure consistency in readings (three consecutive identical readings were considered reliable).

### Immunofluorescence

CFBE41o- wild-type or F508del airway epithelial lines [33], [34] were maintained in Eagle's minimum essential medium (Lonza) with 10% FBS (PAA), 1X L-glutamine and 0.3 mg/ml hygromycin (Invitrogen) at 37°C and 5% CO_2_. Before imaging, cells were seeded on 20 mm diameter cover slips and incubated at 37°C until a confluence of 50-60% was obtained. Fixation was carried out in 4% paraformaldehyde for 10 minutes. After fixation and three 5 minutes washes with PBS-T (1X Phosphate buffered saline with 0.1% Tween), cells were permeabilised with 0.5% Triton X-100 in PBS for 4 minutes. After 5-minutes washes with PBS-T, non-specific binding was prevented by blocking with 2% BSA in 1X PBS, for 30 minutes at room temperature. Primary antibody against calumenin (H40, Santa Cruz) was applied for 2 hours at room temperature. After three 5 minutes washes with PBS-T, cells were incubated with Alexa fluor 488 goat anti-rabbit IgG (Molecular probes) secondary antibody diluted 1∶1000 in 2% BSA for 1 hour 30 minutes. After three 5-minute washes with PBS-T, second labeling against PDI (Protein Disulphide Isomerase, Invitrogen), Golgi marker (abcam ab27043) or EEA1 (BD Biosciences, 610457) was performed using antibody at 1∶1000 concentration in 2% BSA in 1X PBS for 12 hours at 4°C. After three 5-minute washes with PBS-T, cells were incubated with anti-mouse Texas Red (Molecular probes) secondary antibody (1∶1000 dilution in 2% BSA). For CFTR staining, cells were grown on 18 mm coverslips, fixed with 3% paraformaldehyde in TBS for 20 min at room temperature, then rinsed in TBS, permeabilized with 0.10% Triton X-100 in TBS for 10 min, and rinsed again with TBS. The samples were then stained as follows: after fixation, nonspecific sites were blocked with TBS containing 1% goat serum for 60 min at room temperature, then the cells were stained with primary antibody [anti-CFTR antibody H-182 (Santa-Cruz Biotechnology), 1∶100] for 90 min at room temperature and rinsed four times in TBS containing 1% goat serum. Next, samples were incubated with the goat anti-rabbit secondary antibody conjugated to Alexa Fluor 488 (1∶800) for 60 min at room temperature. After five washes with 1% goat serum, second labelling against EEA1 was performed using antibody at 1∶1000 concentration in 1% goat serum for 90 min at room temperature. The cells were then rinsed four times with 1% goat serum and incubated with the goat anti-mouse secondary antibody conjugated to Cy3 (1∶800) for 60 min at room temperature. After five final washes, samples were mounted in VectaShield + DAPI (VECTOR Laboratories Inc., Burlingame, CA), dried, and viewed with AxioStar plus microscope (100X oil, Carl Zeiss GmbH, Jena, Germany). Negative controls were also performed by omitting the primary antibodies. Visualization of all images was performed using Axio Vision Rel. 4.8 software. Pearson's correlation coefficient for colocalisation was estimated using JACoP package on ImageJ [35].

## Results

### Calumenin and other CREC family proteins are significantly more charged and less folded compared to other CFTR associated chaperones

In order to investigate the specific biophysical properties of calumenin as a putative CFTR chaperone, we decided to compare various biophysical parameters such as protein hydropathy (GRAVY), unfoldability, aggregation propensity, charge, instability and disorder of calumenin and other CREC family members with previously described CFTR associated chaperones and calcium binding proteins (See Methods and Table S1). CFTR variants (wild type, F508del, G551D) and intrinsically disordered proteins (IDPs) were also used in this analysis due to their extremely high and low hydropathy values respectively. The high hydrophobicity of CFTR and its mutant sequences is due to the presence of transmembrane segments in their amino acid sequences [36]. IDPs have also been previously characterized by high charge and unfoldedness in their polypeptide sequences [18], [37].

**Figure 1:**
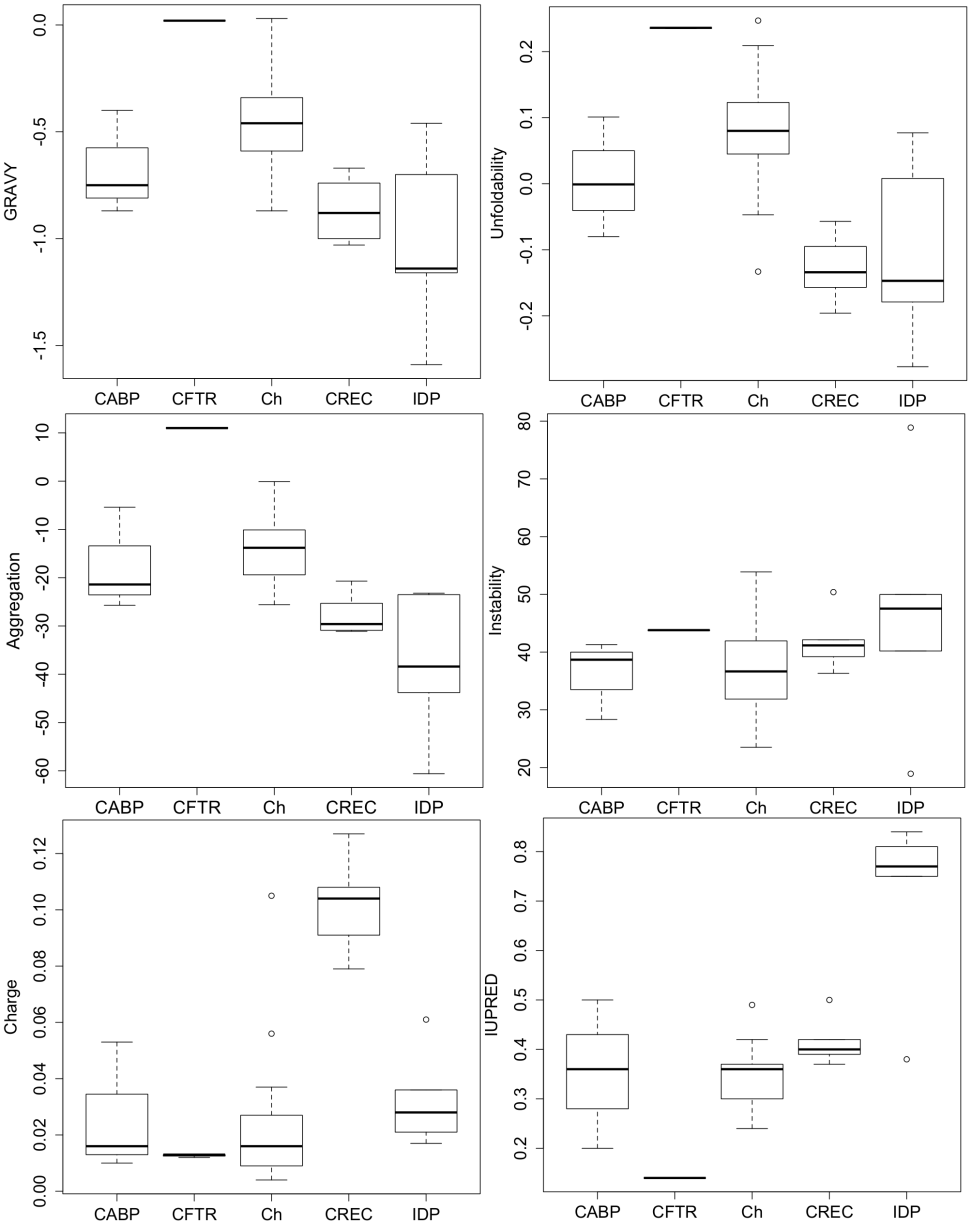
Box-plots showing GRAVY, Unfoldability, Aggregation, Instability, Charge and IUPRED (disorder) measurements. Protein groups used in this analysis are the following: Calcium binding proteins (CABP), CFTR wild-type and mutant (F508del and G551DCFTR) proteins (CFTR), chaperones (Ch), CREC proteins (CREC) and intrinsically disordered proteins (IDP). CREC proteins demonstrated significantly lower hydropathy (GRAVY) scores compared to CFTR proteins (p-value<0.01). These proteins also showed lesser unfoldability compared to CFTR proteins (p-value<0.001) and chaperones (p-value<0.01). In terms of aggregation, CREC proteins posses significantly less proportion of aggregation prone residues in their sequences compared to calcium binding proteins and CFTR proteins (p-values<0.01 and <0.001 respectively). No significant difference between groups was observed for Instability scores. CREC proteins showed a significantly greater amount of charge compared to chaperones (p-value<0.05). IDPs were also significantly more disordered compared to chaperones (p-value<0.05).

As can be observed, a majority of proteins in our dataset demonstrate a GRAVY (grand average hydropathy) score below zero. This score is a useful predictor of protein hydropathy or hydrophobicity, where positive scores indicate that proteins are highly hydrophobic and negative scores reflect protein hydrophilicity. CFTR proteins, being bona fide membrane proteins, demonstrate the highest mean GRAVY scores, while IDPs demonstrate the lowest mean GRAVY scores. An overall significant difference was detected between all the protein classes (ANOVA, F(4,24) = 8.82,p-value<0.001). However, Tukey's post-hoc analysis revealed that CREC proteins, including calumenin, showed significantly lower GRAVY scores compared to CFTR proteins (p-value<0.01), while no significant difference was observed between CREC proteins and other protein classes. Similar analysis was performed for other parameters including unfoldability, aggregation, instability, charge and disorder (IUPRED). Figure 1 depicts the box-plots showing the overall distribution of data belonging to different protein classes for all the biophysical parameters analysed. Significant differences in mean scores were detected for unfoldability (ANOVA, F(4,24) = 9.47,p-value<0.001), aggregation propensity (ANOVA, F(4,24) = 16.31,p<0.001), charge (KW test, Chi-squared value = 13.63, *d.f* = 4.0, p-value<0.01) and disorder (IUPRED) (KW test, Chi-squared value = 17.32, *d.f* = 4.0, p-value<0.01). As can be observed, CREC proteins, including calumenin, were less folded compared to CFTR proteins and chaperones (Tukey's post-hoc test, p-values<0.001 and <0.01 respectively). They were also less aggregation prone compared to CFTR proteins (Tukey's post-hoc test, p-value<0.001). In this respect, they were found to be similar to IDPs, which also showed significantly less aggregation propensity compared to CFTR proteins (Tukey's post-hoc test, p-value <0.001). IDPs were also observed to be significantly more disordered compared to chaperones (Mann-Whitney test with Bonferonni correction, p-value<0.05). Interestingly, we observed that calumenin, along with other CREC proteins possessed a significantly greater amount of charge compared to the chaperone group (Mann-Whitney test with Bonferonni correction, p-value<0.05). No significant difference was observed between different protein groups with respect to their instability values.

**Figure 2:**
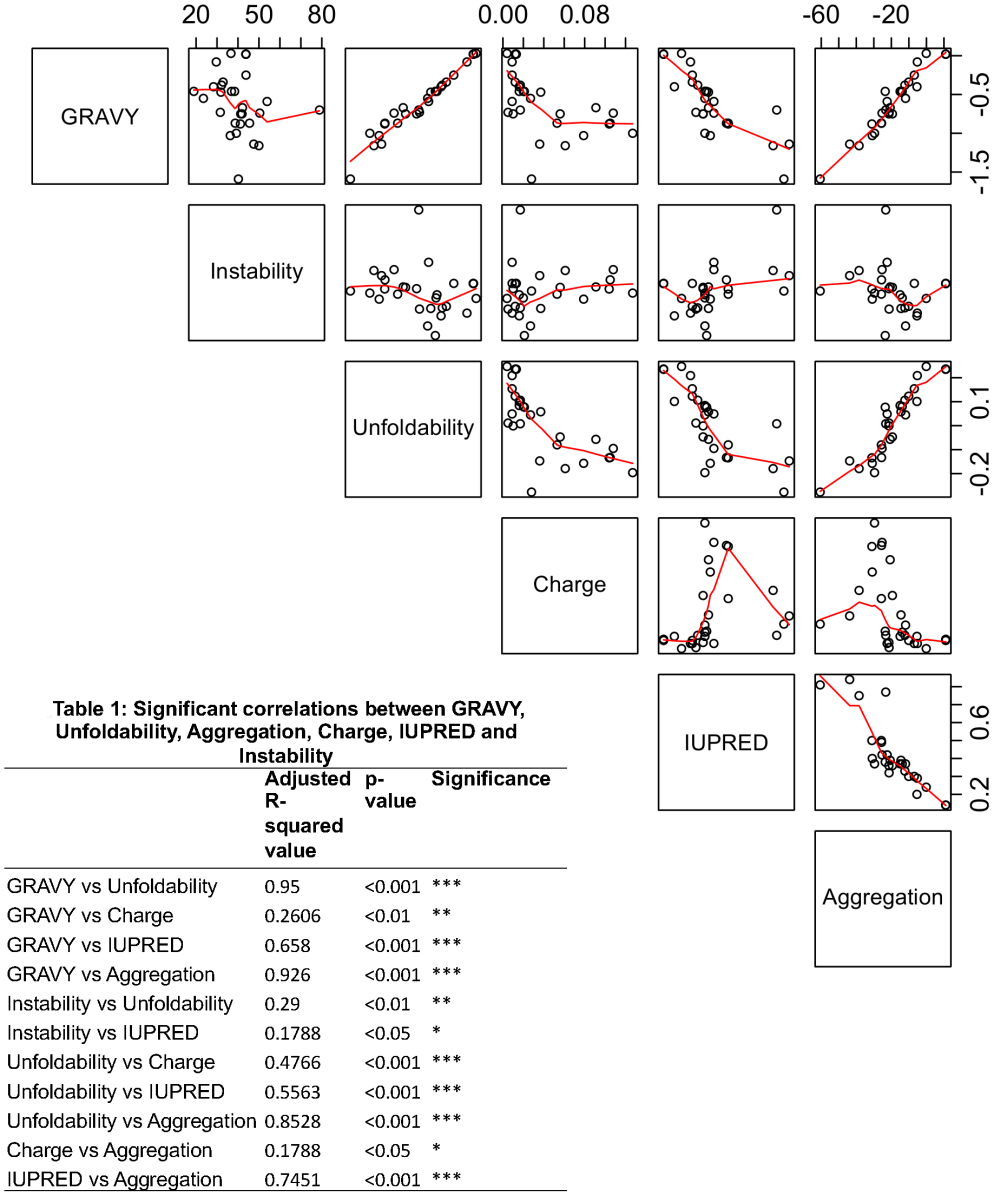
Correlation plots for GRAVY, Unfoldability, Aggregation, Instability, Charge and IUPRED (disorder). In each plot, the y-axis is represented by the score highlighted in each row of the matrix, while it's corresponding x-axis is depicted by parameters described in the rows above or below it. Table 1 shows the pair-wise correlation values for all the parameters analyzed.

All the pair-wise correlation plots are shown in Figure 2. Table 1 (shown in Figure 2) depicts significant pair-wise correlation values observed in our dataset between different biophysical parameters. As can be observed GRAVY scores show a high degree of correlation with protein unfoldability (Adjusted R-squared value: 0.95, p-value<0.001) and aggregation propensity (Adjusted R-squared value:0.93, p-value<0.001). Unfoldability and disorder (IUPRED) were found to be moderately correlated with aggregation propensity (Adjusted R-squared values: 0.85,0.74 respectively, p-values<0.001 and <0.001 respectively). The only variable observed to be weakly correlated with charge was protein unfoldability (Adjusted R-squared value: 0.48, p-value<0.001).

**Figure 3:**
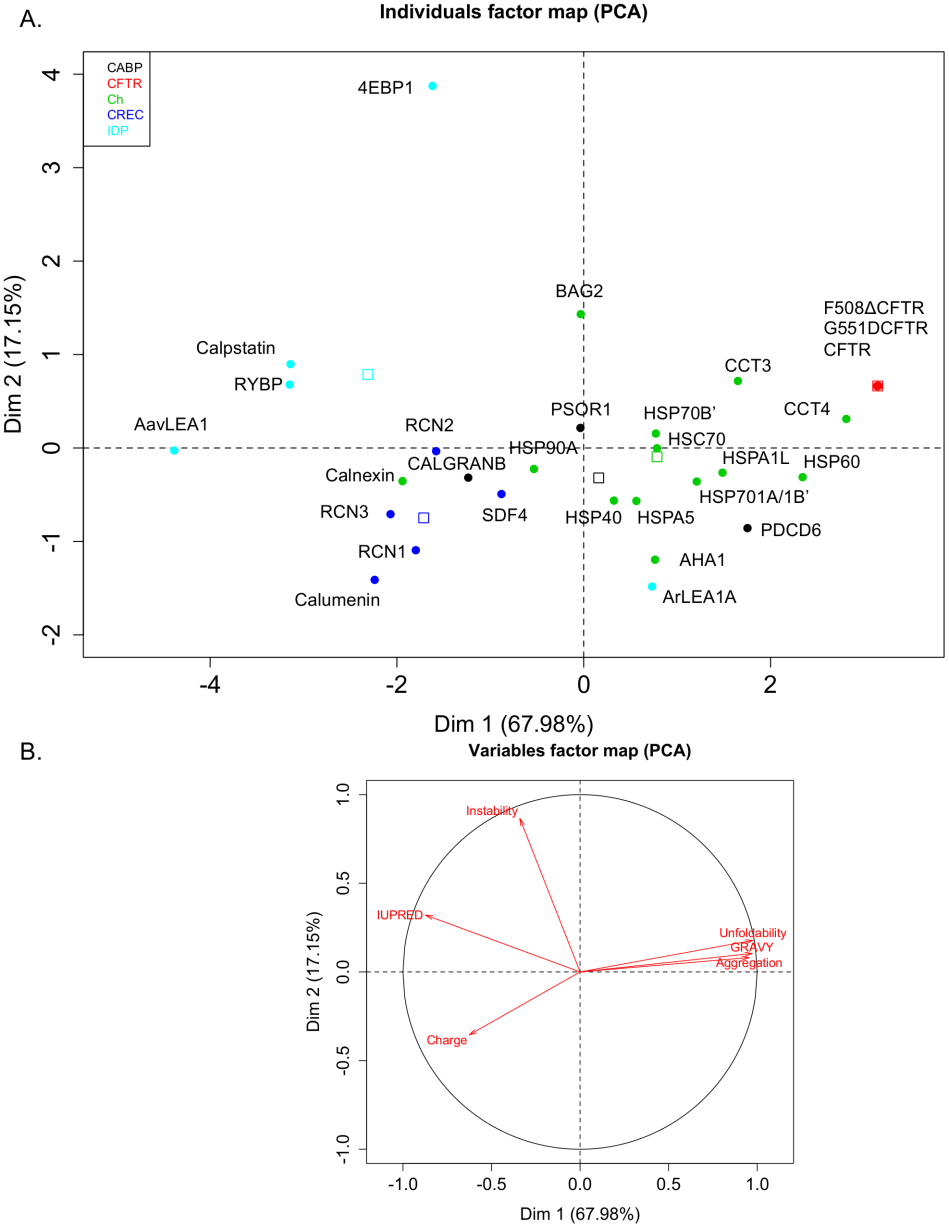
Principal component analysis draws correlations between different biophysical parameters for different protein categories. Multivariate data (Table S1) was scaled and transformed into different components in such a way that the first principal component has the largest possible variance and each succeeding component in turn has the highest variance possible under the constraint that is orthogonal to it (i.e., uncorrelated with) the preceding components. **A.** The first two principal components are represented on the x and y axes respectively and cover 67.98% and 17.15% of the total variance observed in our dataset. **B.** The variables factor map gives directionality to different biophysical parameters as observed on the PCA plot. Each filled circle represents a unique protein. The color legend is described on the top left corner (CABP (black): Calcium binding proteins, CFTR (red): CFTR variants, Ch (green): Chaperones, CREC (blue): CREC proteins, IDP (cyan): intrinsically disordered proteins). As can be observed calumenin lies in the region corresponding to high charge, along with other CREC family members RCN1, RCN2, RCN3 and SDF4. Unfilled open boxes in various colours depict positions of different protein classes on the PCA map.

In order to discern the biophysical landscape of different classes of proteins analysed, multivariate principal component analysis and hierarchial clustering were carried out. As observed in Figure 3A, the first two principal components are represented on the x and y axes respectively and cover 67.98% and 17.15% of the total variance observed in our dataset. The variables factor map (Figure 3B) gives directionality to different biophysical parameters as observed on the PCA plot. As can be observed calumenin lies in the region corresponding to high charge, along with other CREC family members RCN1, RCN2, RCN3 and SDF4. Interestingly, other calcium associated proteins such as calnexin and CALGRANB also cluster in this region. CFTR variants are observed in the region corresponding to high GRAVY, unfoldability and aggregation propensity. Strikingly, we observe a large variation in the physicochemical characteristics of CFTR associated chaperones. Most of the disordered proteins in our dataset lie in the region corresponding to high disorder, except for ArLEA1A, which was observed to cluster with other CFTR associated chaperones on the PCA plot.

**Figure 4:**
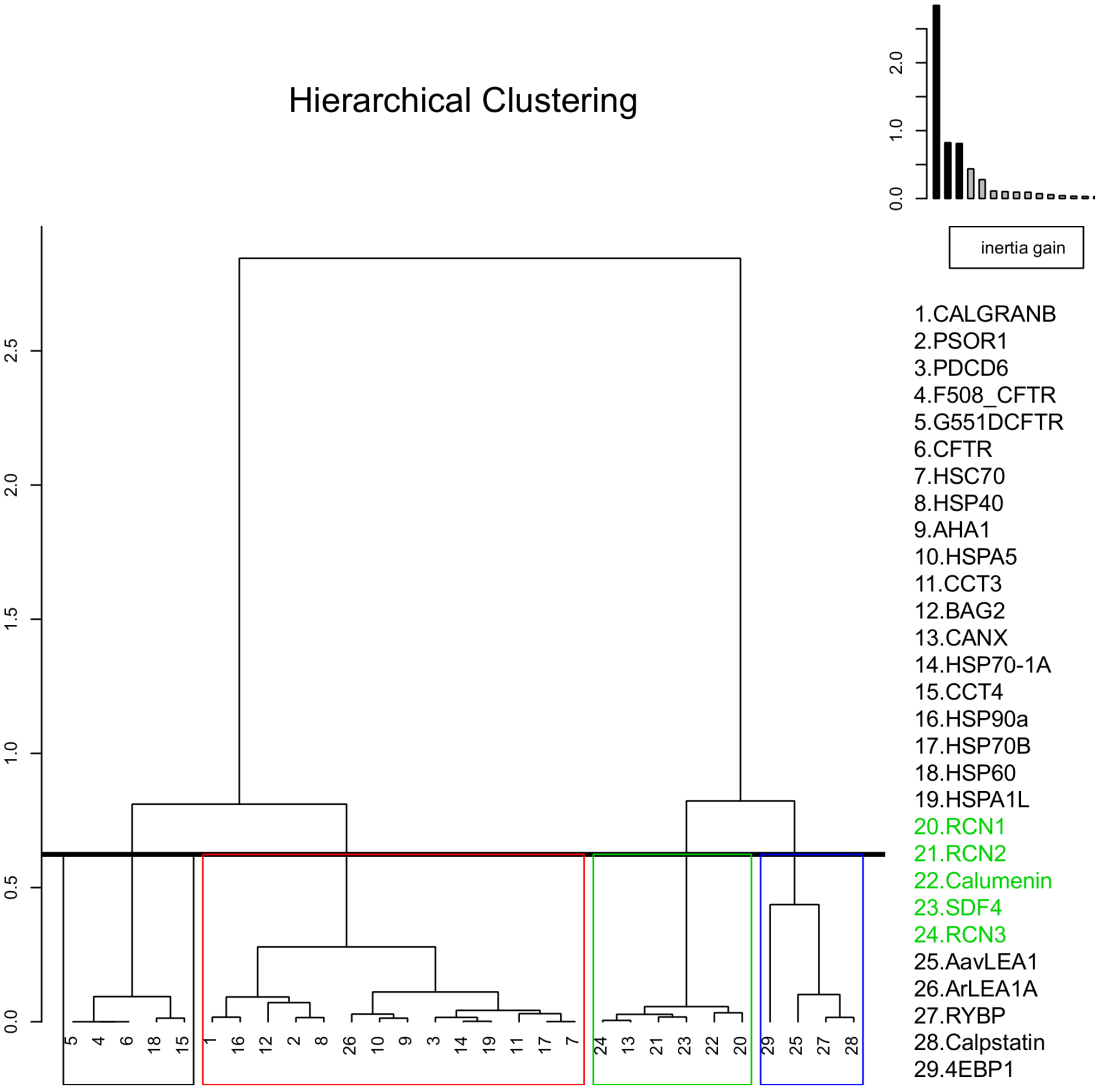
Hierarchial clustering of proteins based on their biophysical features. Sequential clustering was performed by computing the Euclidean distance matrix and by following the Ward's criterion after data transformation during PCA. A dendrogram was drawn, where each vertical line represents a cluster and a horizontal line connecting between any two vertical lines represents the merger of clusters, where its height is related to the dissimilarity measure between the merged clusters. Inertia is defined as multidimensional variance and can be decomposed as variance observed “between” and “within” different clusters, where Ward criterion aims to minimize the increase or “gain” of “within inertia”. As can be observed on the plot on the top right hand corner, a step-wise decrease in inertia was performed until no further decrease was observed for different clusters. CREC proteins are observed in a single cluster (green) along with calnexin, chaperones and calcium binding proteins are observed to be inter-related in a separate cluster (red), while CFTR proteins (black) and disordered proteins cluster separately (blue). CREC proteins are shown in green text.

Hierarchial clustering (Figure 4) further revealed the presence of four related clusters obtained from the factor map, with all the CREC proteins partitioned in a single cluster with calnexin. Calumenin is observed to be most similar to RCN1 protein. Disordered proteins and CFTR variants also cluster separately. Interestingly, we observed considerable similarity between chaperones HSP90A and HSP40 and calcium binding proteins CALGRANB and PSOR1 respectively. Overall, our combined PCA and hierarchial clustering results demonstrate similarity in the biophysical properties of calumenin and other CREC proteins which distinguishes them from other CFTR associated chaperones, disordered proteins and CFTR variants.

### Phylogenetic analysis of CREC proteins reveals common ancestry and similarity with chaperones and calcium binding proteins

**Figure 5:**
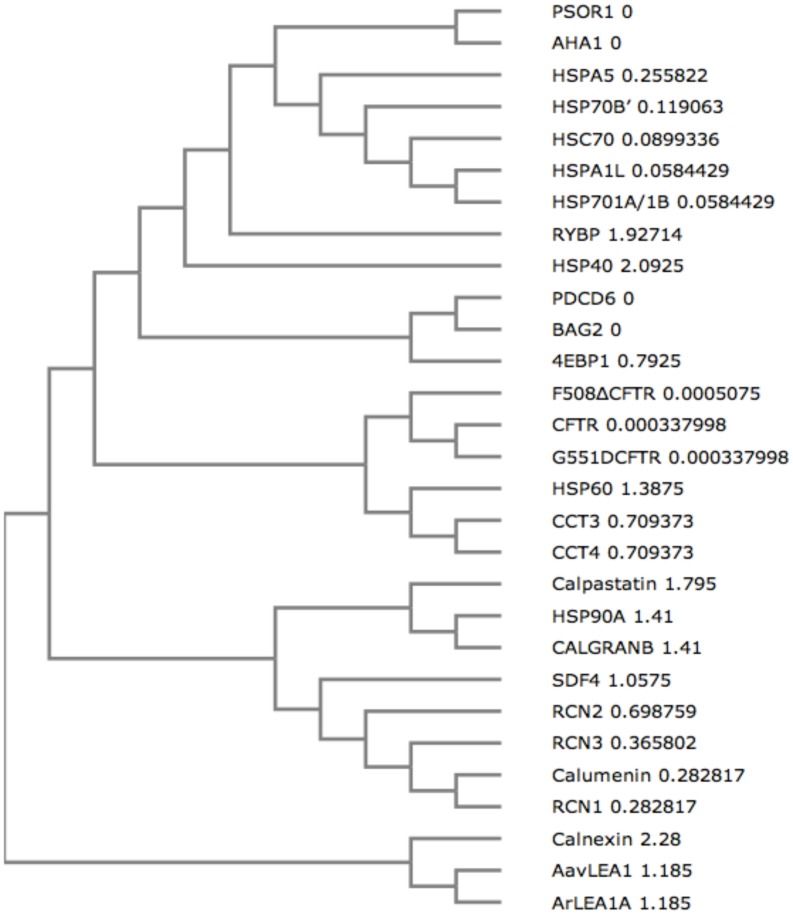
Phylogenetic tree. A cladogram was constructed after multiple sequence alignment of all the protein sequences using Clustal omega. Common evolutionary ancestry is observed for CREC proteins. The evolutionary distance is depicted next to each branch, where 0 indicates collapsing of the two branches into a single node.

In order to establish a relationship between the biophysical properties of CREC proteins, chaperones, calcium binding proteins, IDPs and CFTR variants and their evolutionary relatedness, we aligned their sequences using Clustal omega [29] and constructed a phylogenetic tree using UPGMA clustering method [30], [38]. Figure 5 shows that the CREC proteins share common ancestry and show close relatedness with other chaperones and calcium binding proteins such as HSP90A, Calpastatin and CALGRANB. Calumenin protein is observed to lie closest to RCN1 and RCN3. These results demonstrate correlation between the biophysical properties of calumenin and its phylogeny. Common evolutionary origins are also observed for heat shock family of chaperones (HSPA5, HSPA1L, HSC70 and HSP70), CFTR variants, CCT3/CCT4 and late embryogenesis abundant proteins ArLEA1A/AavLEA1. Interestingly, we observed a close relationship between IDPs (LEA proteins AavLEA1 and ArLEA1A, RYBP, Calpastatin, 4EBP1) and chaperone proteins. These results demonstrate similarity between chaperones and IDP sequences and corroborate previous findings that have shown that chaperones are also characterised by a state of disorder in their protein sequences, which helps in the molecular recognition of their targets [39].

### Calumenin and AavLEA1 reduce F508del-CFTR aggregation

In order to elucidate the specific molecular function of calumenin, an *in vitro* F508del-CFTR folding assay was devised. A similar CFTR-NBD domain folding assay based on the defective folding kinetics of NBD domain has previously shown the anti-aggregation properties of HSC70 as a CFTR chaperone [40]. The principle on which this assay is based is the following. Proteins are initially denatured and refolded in a test tube at 37°C by consecutive addition of GuHCl, a denaturing agent, and a renaturation buffer. In the absence of chaperones, regions of a particular polypeptide sequence containing hydrophobic amino acids like tryptophan undergo ‘hydrophobic collapse’ resulting in the formation of large sized aggregates, which in turn can be monitored by an increase in turbidity at ∼400 nm. Addition of folding factors like chaperones to the reaction mix, can redirect the folding route of a protein by preventing “hydrophobic collapse” and formation of large aggregates.

**Figure 6:**
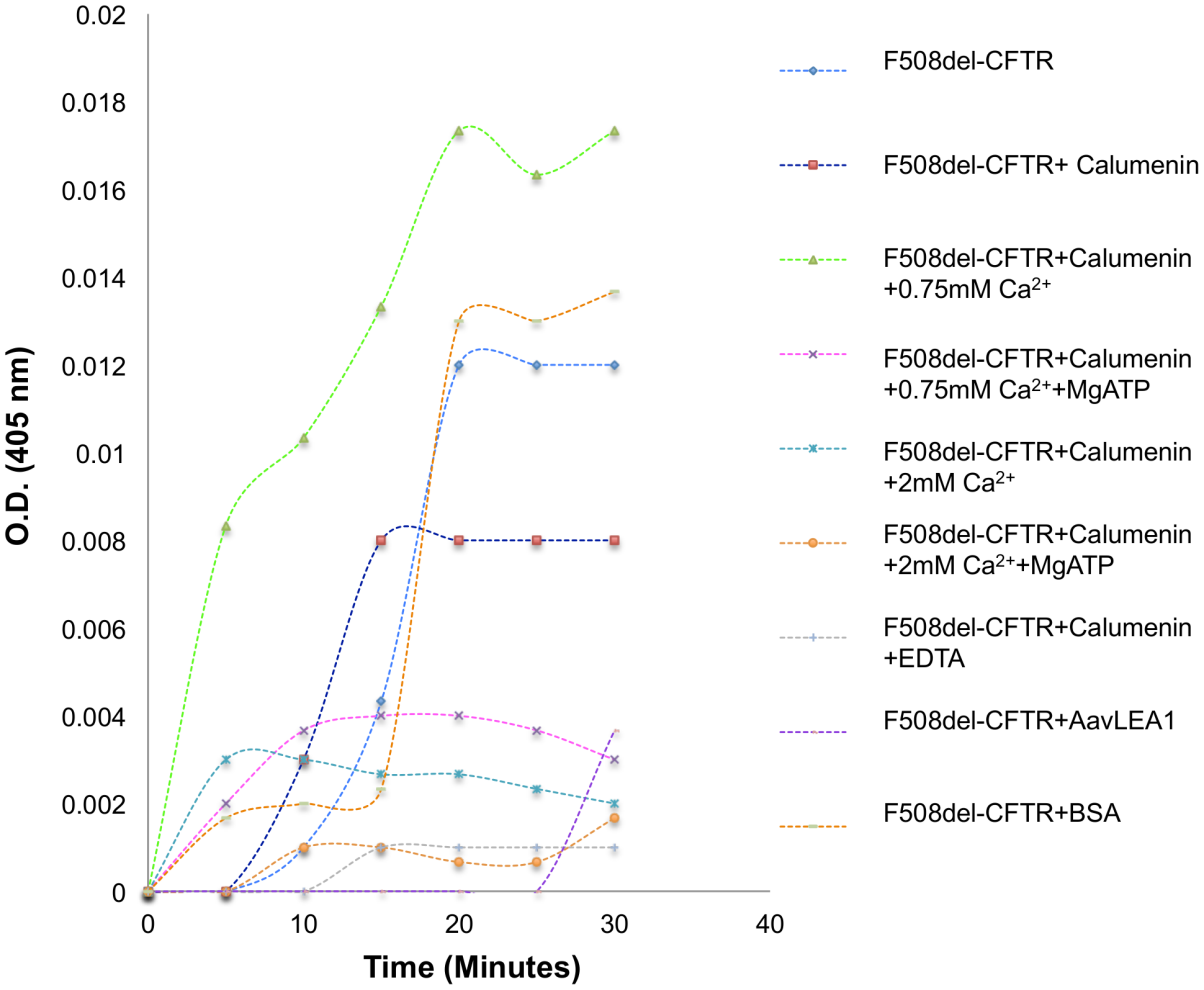
F508del-CFTR folding assay. Proteins were denatured and renatured in a test tube at 37°C resulting in the formation of large sized aggregates whose formation was monitored by an increase in turbidity at 405 nm. F508del-CFTR aggregation was observed to follow a typical ‘S-curve’. Addition of calumenin to the reaction mixture was observed to reduce F508del aggregation by ∼33%. 2 mM calcium further reduced aggregation substantially by ∼83%. In contrast, 0.75 mM calcium was observed to promote aggregation (by ∼44%). Further addition of MgATP, reduced F508del-CFTR aggregation by ∼75% (for 0.75 mM calcium) and ∼86% (for 2 mM calcium). Calumenin with EDTA had the maximum effect (∼92% reduction) in reducing aggregation. AavLEA1, and IDP, prevented F508del-CFTR aggregation at early time points. Bovine serum albumin (BSA) was used as a negative control in our reactions.

We initially performed a test experiment to monitor the kinetics of aggregate formation of purified F508del-CFTR and observed slower aggregation kinetics during the first ten minutes of addition of GuHCl and renaturation buffer at 37°C, followed by an exponential increase in aggregation between 15–20 minutes and saturation between 20–30 minutes. In a control sample without any protein, no exponential increase in aggregation was noted. Following, the initial establishment of this assay, the effect of adding calumenin in a 2∶1 stochiometric ratio (F508del-CFTR: Calumenin) was investigated. As can be observed (Figure 6), calumenin alone was able to reduce F508del aggregation by ∼33%.

Since calumenin is a known calcium binding protein, addition of 2 mM calcium to the reaction mixture was observed to reduce aggregation by ∼83%. In contrast, low calcium (0.75 mM), was observed to promote aggregation by ∼44%. Further addition of MgATP, reduced F508del-CFTR aggregation by ∼75% (for 0.75 mM calcium) and ∼86% (for 2 mM calcium). The most dramatic anti-aggregation effect (∼92% reduction) was observed upon addition of EDTA to the F508del-CFTR and calumenin reaction mixture.

Recently, a family of intrinsically disordered proteins called late embryogenesis abundant (LEA) proteins have been shown to broadly reduce the aggregation of proteins found in anhydrobiotic organisms and of proteins containing polyglutamine repeats [21]. We questioned whether addition of AavLEA1, an LEA protein found in the anhydrobiotic nematode *Aphenlenchus avenae* [41] could also inhibit formation of F508del-CFTR aggregates. As can be observed in Figure 6, addition of AavLEA1 to the folding mix also reduced F508del-CFTR aggregation by ∼69%. These results support previous observations which have described the importance of disorder in chaperone sequences that might play a role in their protein-folding functions.

### Calumenin trafficking is altered in bronchial cells expressing F508del-CFTR

In order to understand the physiological significance of the CFTR-calumenin interaction, we performed intra-cellular localization analysis of calumenin in immortalized human bronchial CFBE41o- epithelial cell line homozygous for F508del mutation or expressing wild-type CFTR. Wild-type CFTR is known to traffic through the ER and the Golgi, to reach the plasma membrane, where its concentration is maintained at a steady state by endocytic recycling through EEA1 containing vesicles, while the F508del mutation is known to increase CFTR retention in the ER and decrease its stability at the membrane by increasing its turn-over through EEA1 vesicles [23], [42], [43].

On the other hand, calumenin and its fifteen protein isoforms of varying lengths, demonstrate complex intra-cellular localization patterns in the secretory pathway, cytoplasm and the nucleus [22], [44]. It is currently not known, whether calumenin, like CFTR, might recycle through EEA1 containing endocytic vesicles as well. Previous research has shown calumenin to localize in the ER in both wild-type and G551DCFTR expressing cells using an antibody towards a single epitope [12], which we now know is present in four out of the fifteen calumenin isoforms- b,3,5 and 13 (See multiple sequence alignment of isoforms in the File S1). Subtle sequence variations of this epitope sequence are also detected in isoforms such as a/c,4,6,7,8,9,10,11,14 and 15 hinting towards significant cross reactivity of this antibody for fourteen out of the fifteen isoforms. However, given the limited availability of antibodies for detection of different isoforms and the technical difficulty involved in raising isoform specific antibodies, we decided to investigate the localization pattern of calumenin and its isoforms in both CFBE41o- wild-type and F508del cells using the antibody currently available to us.

**Figure 7:**
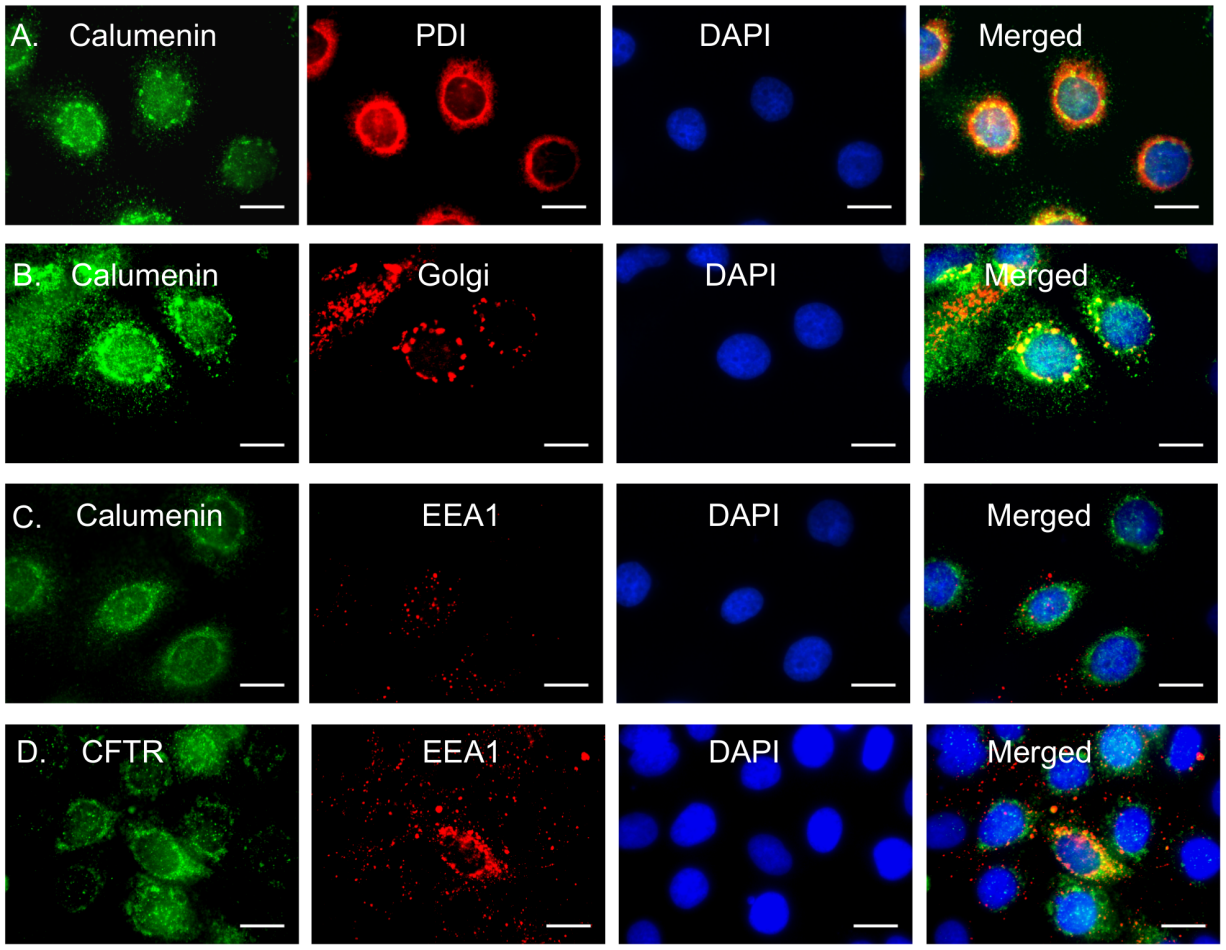
Calumenin trafficking in CFBE41o- cells expressing wild-type CFTR. Calumenin or CFTR (green) in CFBE41o- wild-type or F508del cells were visualized using fluorescence microscopy. Second labeling was performed for either PDI (an ER marker). Golgi marker or EEA1 (marker for endosomal vesicles) (red). The nuclei were stained blue with DAPI. Colocalisation of green and red pixels was detected in merged images (yellow). **A.** Calumenin and PDI (for ER staining) **B.** Calumenin and Golgi and **C.** Calumenin and EEA1 in CFBE41o- wild-type cells. **D.** CFTR and EEA1 in CFBE41o- wild-type cells. Scale bar: 20 µm.

Figure 7 shows calumenin and PDI (ER marker)/Golgi/EEA1(endocytic vesicle marker) staining in CFBE41o- wild type or F508del cells. Individual nuclei were stained using DAPI. CFBE41o- cells were also analyzed for CFTR accumulation in EEA1 vesicles. Table S2 depicts the Pearson's correlation coefficient values between the green and red channels for different intra-cellular markers. In CFBE41o- cells expressing wild-type CFTR, a majority of cells showed vesicular distribution of calumenin (∼75%) indicating its presence in the ER and Golgi compartments, although a few cells also demonstrated its presence in the cytoplasm and nucleus. The Pearson's correlation values observed for calumenin and ER/Golgi markers (Figured 7A and B) were high (0.857 and 0.581 respectively). In contrast, the presence of calumenin in EEA1 vesicles was low (Figure 7C) (Pearson's correlation coefficient: 0.083). CFTR protein showed greater accumulation in EEA1 containing vesicles, compared to calumenin (Figure 7D) (Pearson's correlation coefficient: 0.287). These results are consistent with previous findings, which have also shown the localization of CFTR in EEA1 vesicles [24].

**Figure 8:**
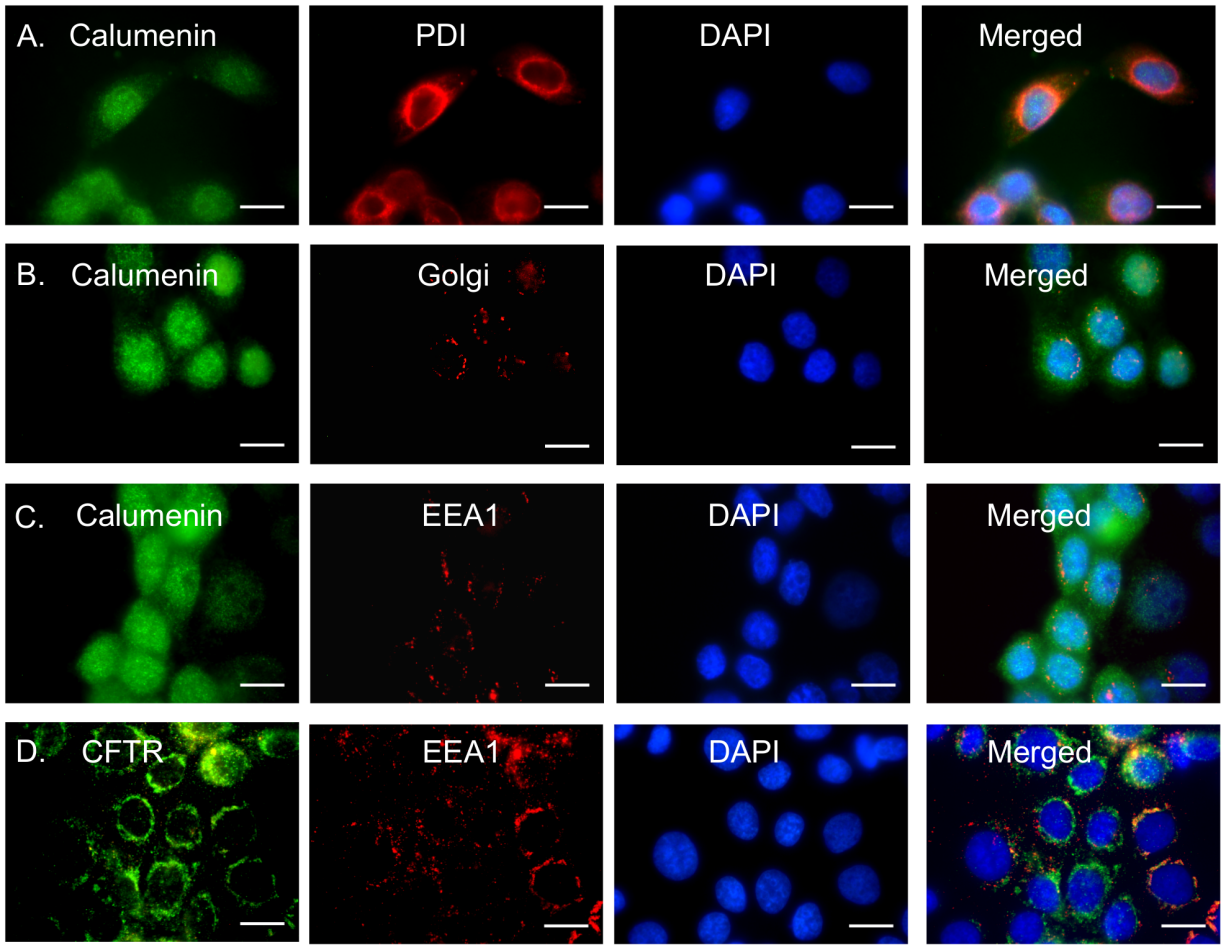
Calumenin trafficking is altered in CFBE41o- cells expressing F508del-CFTR. Calumenin or CFTR (green) in CFBE41o- wild-type or F508del cells were visualized using fluorescence microscopy. Second labeling was performed for either PDI (an ER marker). Golgi marker or EEA1 (marker for endosomal vesicles) (red). The nuclei were stained blue with DAPI. Colocalisation of green and red pixels was detected in merged images (yellow). **A.** Calumenin and PDI (for ER staining) **B.** Calumenin and Golgi and **C.** Calumenin and EEA1 in CFBE41o- cells expressing F508del-CFTR. **D.** CFTR and EEA1 in CFBE41o- cells expressing F508del-CFTR. Scale bar: 20 µm.

In CFBE cells homozygous for the F508del-CFTR mutation, a majority of cells (∼90%) exhibited displacement of calumenin from the secretory pathway to the cytoplasm and the nucleus (Figures 8A–D). This correlated with a marked reduction in the Pearson's correlation coefficient value for calumenin in the Golgi vesicles (0.328), while there was little change observed for its localization in the ER (0.805) (Figures 8A and B), indicating defective trafficking of calumenin through the secretory pathway. Additionally, we also observed enhanced accumulation of both calumenin and CFTR in EEA1 containing endocytic vesicles (Pearson's correlation coefficients: 0.195 and 0.503 respectively)(Figures 8C and D). Overall, our imaging data suggests abnormal trafficking of calumenin isoforms in bronchial cells expressing F508del-CFTR, compared to cells expressing wild-type CFTR. The implications of these findings are further discussed below.

## Discussion

Calumenin is a calcium binding CREC protein with multiple EF-hands that undergoes secretion [22] and has recently been found to be associated with G551DCFTR protein [12]. It has been hypothesized to function as a putative CFTR chaperone, however so far its biophysical properties have remained uncharacterized. In this study we have drawn comparisons between calumenin, including other CREC proteins, and proteins previously known to act as CFTR associated chaperones and calcium binding proteins. The composition of different protein sequences in our dataset has been deduced using various parameters such as hydropathy (GRAVY), unfoldability, aggregation propensity, instability, charge and disorder by applying bioinformatics based prediction tools such as ProtParam [24], FoldIndex [25], IUPRED [26], [27] and Aggrescan [28]. These tools have proved valuable in characterizing the functions of numerous novel proteins in the past [45]–[48].

In our data, we observed high correlation between GRAVY, unfoldability and aggregation scores, however only a moderate correlation was noticed between unfoldability and disorder. Similar correlations between these parameters have been observed in previous studies [45], [46], [49]. Intrinsically disordered proteins (IDPs) [15] were used as a control group in our analysis, since it has been previously proposed that some chaperones are also characterized by a state of disorder [19] and unfoldedness [50], which might help in their molecular recognition properties. For example, Reichmann *et al.* have recently described the unfolded nature of Hsp33 chaperone, an oxidative stress sensor whose unfoldability allows it to stabilize other partially folded client proteins during stressed conditions [50]. It was hypothesized that this comparison might help elucidate whether calumenin and CREC proteins are similar to disordered proteins with respect to their sequence composition.

Our results reveal that calumenin (along with other CREC proteins) is significantly more charged, less folded, hydrophobic and aggregation prone compared to other CFTR associated chaperones. In this respect, its biophysical profile tends to be more closely aligned with IDPs. Our experimental results demonstrating anti-aggregation effects of calumenin and AavLEA1, an IDP belonging to the family of LEA proteins, on F508del-CFTR folding kinetics tend to support this hypothesis. Based on these results, we propose that calumenin might act as a charged, relatively unfolded chaperone of F508del-CFTR. It is possible that it might have an effect on the folding of wild-type CFTR protein as well.

Since calumenin is a known calcium binding protein, similar to other CREC family members, we decided to investigate whether addition of low (0.75 mM) or high amounts of (2 mM) Ca^2+^ ions to the folding mix had any effects on it chaperone activity. Our data showed that a low concentration of Ca^2+^ ions promoted F508del-CFTR aggregation while a higher concentration (2 mM), inhibited aggregation. These results suggest that calumenin could function both as a positive or negative regulator of F508del-CFTR folding and aggregation in the presence of low or high concentrations of Ca^2+^ ions respectively. We can extrapolate our *in vitro* results for calcium dependent F508del-CFTR folding modulation by calumenin, with known *in vivo* changes in ER calcium stores observed in nasal epithelial cells of CF patients. An expansion of the ER and an increased mobility of Ca^2+^ ions in short term (6–11 days) cultures of nasal epithelial cells has been previously reported and is hypothesized to be an adaptive response to chronic infection and inflammation observed in CF patients [51]. In long term cultures (30–40 days), a reversal of this phenotype is observed. One can therefore propose a model whereby calumenin might exert its anti-aggregation effects in the presence of high amounts of Ca^2+^ ions to minimize F508del-CFTR misfolding and aggregation in CF cells during inflammation. However, prolonged inflammation and ER stress are expected to decrease ER calcium levels [52], thereby promoting F508del-CFTR aggregation in the presence of calumenin in the longer term.

The anti-aggregation activity of calumenin was also found to be dependent on the presence of MgATP, where it was observed to synergistically reduce F508del-CFTR aggregation with both low and high amounts of calcium. It is possible that binding of calcium and MgATP to the calumenin-CFTR complex might induce changes in protein conformation that could prevent the ‘hydrophobic collapse’ of F508del-CFTR protein. Indeed, calcium has been shown to induce the α-helical folding and compaction of another CREC protein, RCN1 [53], while.binding of MgATP to the CFTR NBD domain has been known to influence its dimerization and gating function [54].

Lastly, addition of EDTA, a chelating agent, to the folding mix with calumenin, resulted in the most dramatic reduction in F508del-CFTR aggregation kinetics. EDTA is expected to sequester all the positively charged ions such as Mg^2+^ and Ca^2+^ from the reaction mix, thereby allowing calumenin to acquire its native state. Buffer C used in our F508del-CFTR folding assay, indeed contained 2 mM Mg^2+^ ions, which could have inhibited the intrinsic anti-aggregation properties of calumenin, dependent on the high amount of charge in its sequence.

Our *in vitro* aggregation results could be further confirmed by *in vivo* analytical techniques such as fluorescence recovery after photobleaching (FRAP) [55] and Förster resonance energy transfer (FRET) [56] to monitor F508del-CFTR aggregation and interaction with calumenin respectively.

Immunofluorescence imaging of CFBE41o- cells expressing wild-type CFTR revealed that calumenin accumulated in the ER and Golgi compartments as previously reported [22]. This was evident by the high Pearson's correlation coefficient values observed for calumenin and ER/Golgi markers. Interestingly, calumenin was observed to be retro-translocated to the cytoplasm and the nucleus in the majority of CFBE cells expressing F508del-CFTR. We propose there could be three possible explanations for this observed phenomenon. (1) This might be a consequence of the unfolded protein response [57] triggered in cells expressing misfolded proteins that allows them to be cleared by the ER associated degradation machinery [58]. (2) Calumenin might be mobilized from the ER in response to intra-cellular Ca^2+^ signaling, that has been found to be increased in cells expressing F508del-CFTR [59]. (3) Calumenin might undergo alternative splicing into various isoforms that might in turn translocate to the cytoplasm and nucleus [22], [44]. Alternative splicing of XBP1 transcript mediated by inositol receptor endonuclease 1 (IRE1) has been shown to be triggered during the unfolded protein response triggered in response to misfolded proteins in the endoplasmic reticulum [60]. Calumenin isoforms could also be potentially phosphorylated and translocated into the nucleus [44] where they might elicit changes in gene expression. Previously it has been shown that calreticulin, another calcium sensitive chaperone in the ER, can affect the protein levels of myocyte enhancer factor (MEF) 2C, a cardiac specific transcription factor involved in cardiac development [61]. We can also expect significant heterogeneity in distribution of various calumenin isoforms in human bronchial cells as has been demonstrated for a number of calcium regulated proteins in the endoplasmic reticulum [62].

Since CFTR is maintained at a steady state in the plasma membrane by endocytic recycling through EEA1 containing vesicles and the F508del mutation is known to decrease its stability at the membrane by increasing its turn-over through these vesicles [23], [42], [43], it was decided to compare endocytic trafficking of calumenin in both the wild-type CFTR and F508del-CFTR expressing CFBE41o- cells. It was hypothesized that if the calumenin-CFTR interaction was strong, we would expect equal localization of calumenin and CFTR in EEA1 vesicles.

Our data revealed that calumenin localization in EEA1 containing vesicles was substantially reduced compared to CFTR in both wild-type and F508del cells. These results suggest that calumenin, might not be as efficiently recycled in the endocytic pathway, as compared to CFTR. We speculate that the low pH of the secretory vesicles (∼5.5) compared to the ER (∼7) might result in conformational changes of calumenin, which has an isoelectric point (pI) of ∼4.4, allowing it to detach from the CFTR complex and undergo further processing required for its function as a secretory protein. The low pH of the secretory vesicles is known to induce conformational changes in protein structures resulting in the formation of aggregates [63] or novel protein-protein interactions required for vesicular sorting [64]. However, in cells expressing F508del-CFTR, calumenin showed increased accumulation in EEA1 vesicles, suggesting its increased endocytic uptake in CF cells.

Currently, it is not clear how calumenin-CFTR interaction might contribute towards the observed pathophysiology of cystic fibrosis. On one hand, we can speculate that calumenin might prevent deleterious misfolding and aggregation of F508del-CFTR, and possibly G551DCFTR, to a certain extent, as part of the cell's innate defense mechanism in the short term. However, given that we observe a translocation of calumenin in the cytoplasm and the nucleus in a majority of F508del-CFTR expressing cells, indicates that its potentially beneficial effect in regulating F508del-CFTR folding might be compromised due to its displacement from the ER in the longer term. Previously, it has been suggested that depletion of ER calcium by inhibitors of calcium pumps such as thapsigargin, allows misfolded F508del-CFTR to proceed to the membrane more efficiently by releasing it from its associated calcium dependent chaperones [65]. We speculate that calumenin might also inhibit F508del-CFTR trafficking in a manner similar to other calcium dependent chaperones and therefore its association with CFTR might be deleterious to the cell.

Given the paucity of cellular chaperones (including calumenin) in countering the negative effects of F508 deletion in CF patients, one can envisage designing superior synthetic peptides with optimized biophysical parameters that might be able to structurally correct the F508del folding defect. Since calumenin has been characterized as a charged, F508del-CFTR folding modulator in this paper, optimising the quantity of charge in peptide sequences, could be one strategy to induce correct folding of protein sequences. Further insights into the design parameters for such peptides are gained by the observation that certain suppressor mutations such as G550E and I539T can partially rescue the F508del-CFTR to the cell surface [6]. Both these mutations involve a substitution of a polar amino acid with a hydrophobic amino acid. One can imagine that it might be possible to design peptides with optimal hydrophobic content in their sequence composition and specificity towards the NBD1 domain to correct the F508del-CFTR folding defect during the early stages protein translation in the ER in a similar manner [66].

An alternative strategy could involve designing peptides that might prevent the association of chaperones with mutant CFTR, thereby enabling it to traffic more efficiently through the secretory pathway. A similar chaperone displacement strategy has been previously applied by expressing the NBD1 plus the regulatory domain fragment in human airway cells [67] which partially restored F508del-CFTR trafficking and functions. Increasing the amount of disorder in peptide sequences could also prevent deleterious chaperone associations with F508del-CFTR. Additionally, such peptides could potentially also serve as synthetic “molecular shields” [16] by preventing the aggregation of misfolded mutant CFTR molecules. Our results regarding the anti-aggregation activity of AavLEA1, an IDP, lend further support to this hypothesis. Bioinformatics based selection and screening of combinatorial peptide libraries [68], [69] could be potentially applied to synthesize peptides with maximum structural correction efficiencies of mutant CFTR proteins. It would be exciting to test this approach in the future as a potential therapeutic strategy in CF.

## Supporting Information

Table S1 GRAVY, Instability, Unfoldability, Charge, IUPRED and Aggregation scores of proteins used in our analysis.
(XLS)

Table S2 Pearson's correlation coefficient values between red and green channels.
(XLS)

File S1 Multiple sequence alignment of calumenin isoforms.
(DOC)
