# Correction: Biophysical Characterisation of Calumenin as a Charged F508del-CFTR Folding Modulator

**DOI:** 10.1371/journal.pone.0110572

**Published:** 2014-10-15

**Authors:** The PLOS ONE Staff

The affiliation for the third author is incorrect. Bridget Culleton is not affiliated with #5 but with #4 Department of Biology, National University of Ireland, Maynooth, Maynooth, Co Kildare, Ireland.

The Publisher apologizes for this error.
