# Supplementary material for: Biophysical Characterisation of Calumenin as a Charged F508del-CFTR Folding Modulator
Source: PLoS One. 2014 Aug 13;9(8):e104970. doi: 10.1371/journal.pone.0104970 (PMC4132023; doi:10.1371/journal.pone.0104970)
Supplement: File S1 — Multiple sequence alignment of calumenin isoforms. (DOC) [file pone.0104970.s003.doc]

Supplementary Information

Clustal 2.1 Multiple Sequence Alignment of Calumenin Isoforms

H-40 (calumenin antibody, Santa Cruz) epitope is highlighted in yellow.

**Protein ID** Corresponding Calumenin Isoform

| EAW83676.1 | isoform a/c |
| --- | --- |
| EAW83677.1 | isoform b |
| ADG45004.1 | isoform 3 |
| ADG45005.1 | isoform 4 |
| ADG45006.1 | isoform 5 |
| ADG45007.1 | isoform 6 |
| ADG45008.1 | isoform 7 |
| ADG45009.1 | isoform 8 |
| ADG45010.1 | isoform 9 |
| ADG45011.1 | isoform 10 |
| ADG45012.1 | isoform 11 |
| ADG45013.1 | isoform 12 |
| ADG45014.1 | isoform 13 |
| ADG45015.1 | isoform 14 |
| ADG45016.1 | isoform 15 |

CLUSTAL 2.1 multiple sequence alignment

gi|295848269|gb|ADG45015.1| VHNDAQSFDYDHDAFLGAEEAK----TFDQLT--PEESKERLGKIVSKID 89

gi|295848267|gb|ADG45014.1| VHNDAQSFDYDHDAFLGAEEAK----TFDQLT--PEESKERLGKIVSKID 81

gi|295848261|gb|ADG45011.1| VHNDAQSFDYDHDAFLGAEEAK----TFDQLT--PEESKERLGKIVSKID 81

gi|295848263|gb|ADG45012.1| VHNDAQSFDYDHDAFLGAEEAK----TFDQLT--PEESKERLGMIVDKID 81

gi|295848255|gb|ADG45008.1| VHNDAQSFDYDHDAFLGAEEAK----TFDQLT--PEESKERLGMIVDKID 81

gi|295848257|gb|ADG45009.1| VHNDAQSFDYDHDAFLGAEEAK----TFDQLT--PEESKERLGMIVDKID 81

gi|295848265|gb|ADG45013.1| VHNDAQSFDYGHDAFLGAEEAK----TFDQLT--PEESKERLG------- 74

gi|295848253|gb|ADG45007.1| VHNDAQSFDYDHDAFLGAEEAK----TFDQLT--PEESKERLGKIVSKID 81

gi|295848247|gb|ADG45004.1| VHNDAQSFDYDHDAFLGAEEAK----TFDQLT--PEESKERLGKIVSKID 89

gi|295848259|gb|ADG45010.1| VHNDAQSFDYDHDAFLGAEEAK----TFDQLT--PEESKERLGKIVSKID 81

gi|295848251|gb|ADG45006.1| VHNDAQSFDYDHDAFLGAEEAK----TFDQLT--PEESKERLGKIVSKID 81

gi|119604083|gb|EAW83677.1| VHNDAQSFDYDHDAFLGAEEAK----TFDQLT--PEESKERLGKIVSKID 81

gi|295848271|gb|ADG45016.1| VQETMEDIDKNADGFIDLEEYIGDMYSHDGNTDEPEWVKSEREQFVEFRD 91

gi|119604082|gb|EAW83676.1| VQETMEDIDKNADGFIDLEEYIGDMYSHDGNTDEPEWVKTEREQFVEFRD 242

gi|295848249|gb|ADG45005.1| VQETMEDIDKNADGFIDLEEYIGDMYSHDGNTDEPEWVKTEREQFVEFRD 250

*:: :.:* . *.*:. ** :.* * ** * .

gi|295848269|gb|ADG45015.1| GDKDGFVTVDELK------------------------------------- 102

gi|295848267|gb|ADG45014.1| GDKDGFVTVDELK------------------------------------- 94

gi|295848261|gb|ADG45011.1| GDKDGFVTVDELK------------------------------------- 94

gi|295848263|gb|ADG45012.1| ADKDGFVTEGELK------------------------------------- 94

gi|295848255|gb|ADG45008.1| ADKDGFVTEGELK------------------------------------- 94

gi|295848257|gb|ADG45009.1| ADKDGFVTEGELK------------------------------------- 94

gi|295848265|gb|ADG45013.1| --------------------------------------------------

gi|295848253|gb|ADG45007.1| GDRDGFVTVDELK------------------------------------- 94

gi|295848247|gb|ADG45004.1| GDKDGFVTVDELK------------------------------------- 102

gi|295848259|gb|ADG45010.1| GDKDGFVTADELK------------------------------------- 94

gi|295848251|gb|ADG45006.1| GDKDGFVTVDELKDWIKFAQKRWIYEDVERQWKGHDLNEDGLVSWEEYKN 131

gi|119604083|gb|EAW83677.1| GDKDGFVTVDELK------------------------------------- 94

gi|295848271|gb|ADG45016.1| KNRDGKMDKEETK------------------------------------- 104

gi|119604082|gb|EAW83676.1| KNRDGKMDKEETK------------------------------------- 255

gi|295848249|gb|ADG45005.1| KNRDGKMDKEETK------------------------------------- 263

gi|295848269|gb|ADG45015.1| --------------------------------------DWIKSAQKRWIY 114

gi|295848267|gb|ADG45014.1| --------------------------------------DWIKFAQKRWIY 106

gi|295848261|gb|ADG45011.1| --------------------------------------DWIKFAQKRWIY 106

gi|295848263|gb|ADG45012.1| --------------------------------------SWIKHAQKKYIY 106

gi|295848255|gb|ADG45008.1| --------------------------------------SWIKHAQKKYIY 106

gi|295848257|gb|ADG45009.1| --------------------------------------SWIKHAQKKYIY 106

gi|295848265|gb|ADG45013.1| --------------------------------------------------

gi|295848253|gb|ADG45007.1| --------------------------------------DWIKFAQKRWIY 106

gi|295848247|gb|ADG45004.1| --------------------------------------DWIKFAQKRWIY 114

gi|295848259|gb|ADG45010.1| --------------------------------------DWILPSDYDHAE 106

gi|295848251|gb|ADG45006.1| ATYGYVLDDPDPDDGFNYKQMMVRDERRFKMADKEETKDWILPSDYDHAE 181

gi|119604083|gb|EAW83677.1| --------------------------------------DWIKFAQKRWIY 106

gi|295848271|gb|ADG45016.1| --------------------------------------DWILPSDYDHAE 116

gi|119604082|gb|EAW83676.1| --------------------------------------DWILPSDYDHAE 267

gi|295848249|gb|ADG45005.1| --------------------------------------DWILPSDYDHAE 275

gi|295848269|gb|ADG45015.1| EDVERQWKGHDLNEDGLVSWEEYKNATYGYVLE----------------- 147

gi|295848267|gb|ADG45014.1| EDVERQWKGHDLNEDGLVSWEEYKNATYGYVLE----------------- 139

gi|295848261|gb|ADG45011.1| EDVERQWKGHDHNEDGLVSWEEYKNATYGYVLDDPDPDDGFNYKQMMVRD 156

gi|295848263|gb|ADG45012.1| DNVENQWQEFDMNQDGLISWDEYRNVTYGTYLDDPDPDDGSNYKQMMVRD 156

gi|295848255|gb|ADG45008.1| DNVENQWQEFDMNQDGLISWDEYRNVTYGTYLDDPDPDDGFNYKQMMG-- 154

gi|295848257|gb|ADG45009.1| DNVENQWQEFDMNQDGLISWDEYRNVTYGTYLDDPDPDDGFNYKQMMVRD 156

gi|295848265|gb|ADG45013.1| --------------------------------------------------

gi|295848253|gb|ADG45007.1| EDVERQWKGHGLNEDGLVSWEEYKNATYGYVLDDPDPDDGFNYKQMMVRD 156

gi|295848247|gb|ADG45004.1| EDVERQWKGHDLNEDGLVSWEEYKNATYGYVLDDPDPDDGFNYKQMMVRD 164

gi|295848259|gb|ADG45010.1| AEARHLVYESDQNKDGKLTKEEIVDK-YDLFVGSQATDFG---EALVRHD 152

gi|295848251|gb|ADG45006.1| AEARHLVYESDQNKDGKLTKEEIVDK-YDLFVGSQATDFG---EALVRHD 227

gi|119604083|gb|EAW83677.1| EDVERQWKGHDLNEDGLVSWEEYKNATYGYVLDDPDPDDGFNYKQMMVRD 156

gi|295848271|gb|ADG45016.1| AEARHLVYESDQNKDGKLTKEEIVDK-YDLFVGSQATDFG---EALVRHD 162

gi|119604082|gb|EAW83676.1| AEARHLVYESDQNKDGKLTKEEIVDK-YDLFVGSQATDFG---EALVRHD 313

gi|295848249|gb|ADG45005.1| AEARHLVYESDQNKDGKLTKEEIVDK-YDLFVGSQATDFG---EALVRHD 321

gi|295848269|gb|ADG45015.1| --------------------------------------------------

gi|295848267|gb|ADG45014.1| --------------------------------------------------

gi|295848261|gb|ADG45011.1| ERRFKMADKDGDLIATKEEFTAFLHPEEYDYMKDIVVQETMEDIDKNADG 206

gi|295848263|gb|ADG45012.1| ERRFKMADRARAVC------------------------------------ 170

gi|295848255|gb|ADG45008.1| ---ILMSRNG---------------------------------------- 161

gi|295848257|gb|ADG45009.1| ERRFKMADKDGDLIATKEEIVDKYDLFVGSQATDFGEALVRHDEF----- 201

gi|295848265|gb|ADG45013.1| --------------------------------------------------

gi|295848253|gb|ADG45007.1| ERRFKMADKDGDLIATKEEFTAFLHPEEYDYMKDIVVQETMEDIDKNADG 206

gi|295848247|gb|ADG45004.1| ERRFKMADKDGDLIATKEEFTAFLHPEEYDYMKDIVVQETMEDIDKNADG 214

gi|295848259|gb|ADG45010.1| EF------------------------------------------------ 154

gi|295848251|gb|ADG45006.1| EF------------------------------------------------ 229

gi|119604083|gb|EAW83677.1| ERRFKMADKDGDLIATKEEFTAFLHPEEYDYMKDIVVQETMEDIDKNADG 206

gi|295848271|gb|ADG45016.1| EF------------------------------------------------ 164

gi|119604082|gb|EAW83676.1| EF------------------------------------------------ 315

gi|295848249|gb|ADG45005.1| EF------------------------------------------------ 323

gi|295848269|gb|ADG45015.1| --------------------------------------------------

gi|295848267|gb|ADG45014.1| --------------------------------------------------

gi|295848261|gb|ADG45011.1| FIDLEEYIGWQAYQGGDR-------------------------------- 224

gi|295848263|gb|ADG45012.1| --------------------------------------------------

gi|295848255|gb|ADG45008.1| --------------------------------------------------

gi|295848257|gb|ADG45009.1| --------------------------------------------------

gi|295848265|gb|ADG45013.1| --------------------------------------------------

gi|295848253|gb|ADG45007.1| FIDLEEYIGDMYSHDGNTDESDQNKDGKLTKEEIVDKYDLFVGSQATDFG 256

gi|295848247|gb|ADG45004.1| FIDLEEYIGDMYSHDGNTDEPEWVKTEREQFVEFRDKNRDGKMDKEETKD 264

gi|295848259|gb|ADG45010.1| --------------------------------------------------

gi|295848251|gb|ADG45006.1| --------------------------------------------------

gi|119604083|gb|EAW83677.1| FIDLEEYIGDMYSHDGNTDEPEWVKTEREQFVEFRDKNRDGKMDKEETKD 256

gi|295848271|gb|ADG45016.1| --------------------------------------------------

gi|119604082|gb|EAW83676.1| --------------------------------------------------

gi|295848249|gb|ADG45005.1| --------------------------------------------------

gi|295848269|gb|ADG45015.1| --------------------------------------------------

gi|295848267|gb|ADG45014.1| --------------------------------------------------

gi|295848261|gb|ADG45011.1| --------------------------------------------------

gi|295848263|gb|ADG45012.1| --------------------------------------------------

gi|295848255|gb|ADG45008.1| --------------------------------------------------

gi|295848257|gb|ADG45009.1| --------------------------------------------------

gi|295848265|gb|ADG45013.1| --------------------------------------------------

gi|295848253|gb|ADG45007.1| EALVRHDEF----------------------------------------- 265

gi|295848247|gb|ADG45004.1| WILPSDYDHAEAEARHLVYESDQNKDGKLTKEEIVDKYDLFVGSQATDFG 314

gi|295848259|gb|ADG45010.1| --------------------------------------------------

gi|295848251|gb|ADG45006.1| --------------------------------------------------

gi|119604083|gb|EAW83677.1| WILPSDYDHAEAEARHLVYESDQNKDGKLTKEEIVDKYDLFVGSQATDFG 306

gi|295848271|gb|ADG45016.1| --------------------------------------------------

gi|119604082|gb|EAW83676.1| --------------------------------------------------

gi|295848249|gb|ADG45005.1| --------------------------------------------------

gi|295848269|gb|ADG45015.1| ---------

gi|295848267|gb|ADG45014.1| ---------

gi|295848261|gb|ADG45011.1| ---------

gi|295848263|gb|ADG45012.1| ---------

gi|295848255|gb|ADG45008.1| ---------

gi|295848257|gb|ADG45009.1| ---------

gi|295848265|gb|ADG45013.1| ---------

gi|295848253|gb|ADG45007.1| ---------

gi|295848247|gb|ADG45004.1| EALVRHDEF 323

gi|295848259|gb|ADG45010.1| ---------

gi|295848251|gb|ADG45006.1| ---------

gi|119604083|gb|EAW83677.1| EALVRHDEF 315

gi|295848271|gb|ADG45016.1| ---------

gi|119604082|gb|EAW83676.1| ---------

gi|295848249|gb|ADG45005.1| ---------
